# *Syzygium aromaticum* Essential Oil as a Safe Natural Solution to Control Bacteria in Hatching Eggs

**DOI:** 10.3390/pathogens14050422

**Published:** 2025-04-27

**Authors:** Gabriel da Silva Oliveira, Concepta McManus, Vinícius Machado dos Santos

**Affiliations:** 1 Faculty of Agronomy and Veterinary Medicine, University of Brasília, Brasília 70910-900, Brazil; 2 Center for Nuclear Energy in Agriculture (CENA), University of São Paulo, São Paulo 13416-000, Brazil; 3 Laboratory of Poultry Science, Federal Institute of Brasília—Campus Planaltina, Brasília 73380-900, Brazil

**Keywords:** aromatic plants, chick embryo, hatching eggs, poultry antibacterials, sanitizers

## Abstract

The bacterial contamination of eggshells may be responsible for embryonic mortality, as may the contamination of chicks and broilers. Poor incubation results may be related to high levels of contamination that overcome the antibacterial defenses of the eggshell structure and the poultry’s immune system. Although synthetic antibacterial formulations have demonstrated efficacy in controlling eggshell bacteria, they have also demonstrated toxicity to the embryo. In this study, we aimed to establish a safe and antibacterial topical protocol using *Syzygium aromaticum* essential oil (SAEO) to sanitize hatching chicken eggs. We evaluated the antibacterial capacity of this essential oil and used the ‘hen’s egg test on chorioallantoic membrane’ (HET-CAM) model to measure its toxicity. Our results demonstrated the high effectiveness of SAEO in reducing the bacterial load on eggshells, suggesting that this natural compound is a promising egg sanitizer. However, although the HET-CAM model did not indicate signs of toxicity for pure SAEO, we recommend that its application, following dilution in grain alcohol, be carried out on the surface of eggshells and never directly in the internal embryonic compartment, owing to the toxicity of alcohol.

## 1. Introduction

Pathogenic bacterial contamination in poultry can lead to septicemia, with necrosis and purulent exudate in the lungs, as well as systemic alterations such as hepatosplenomegaly, splenomegaly, renomegaly, and generalized congestion [1]. Additionally, bacterially contaminated eggs may exhibit darkened yolks and liquefied albumen with a grayish coloration [2], while contaminated meat may emit pungent, fishy, rotten-egg, and ammonia-like odors [3]. Surprisingly, the contaminations mentioned earlier can originate from the eggshell itself, even though this may seem unlikely at first glance. We can imagine a situation where chicks hatch from eggs whose shells are contaminated with pathogenic bacteria. During hatching, these bacteria come into direct contact with the chicks, which can become hosts in the early stages of life. Once taken to the farms, these animals can spread the bacteria, contaminating other poultry. This can result in immediate impacts, such as the death of the chicks, or more silent outbreaks, with poultry remaining asymptomatic until adulthood but continuing to spread the bacteria through poultry products. The possibility of this happening is not minimal if basic sanitary control practices are not carried out.

Controlling the bacterial load on eggshells can be a decisive action to prevent the spread of bacteria in hatcheries and farms, as well as to increase the survival prospects of embryos until adulthood, ensuring their health and the quality of poultry products. This need has led to research into poultry sanitizers, especially for hatching eggs, resulting in the creation of antibacterial plans that employ synthetic and natural sanitizers [4,5,6,7,8,9,10,11,12,13]. The development of poultry sanitizers that are effective and free of undesirable characteristics remains an unmet challenge, and unfortunately, this development may continue. However, sanitizers for hatching eggs must be effective at destroying microorganisms, particularly bacteria, while causing minimal disruption to the living organisms involved in the process [14]. Regarding safe products, there is a strong preference for considering compounds derived from natural sources, such as plants, as opposed to synthetic products, which are often associated with toxicities. Designing customized antibacterial sanitizers with plant-derived active components is a desirable approach for topical application on the surface of eggshells [5,11,12,13,15,16].

Routine antibacterial management for hatching eggs with essential oils has been suggested [17,18,19]. *Syzygium aromaticum* (clove) is an aromatic plant that can reach a height of up to 11.72 m, with a stem circumference of 115.55 cm. The dried clove buds can have a moisture content of 6.82% and an essential oil content of 17.96%. This essential oil, which can be composed of more than 70% eugenol [20], exhibits remarkable antibacterial properties against a wide range of bacteria, including *Acinetobacter baumannii*, *Pseudomonas aeruginosa*, *Staphylococcus aureus*, and *Enterococcus faecalis* [21]. A recently published review reported that clove essential oil is a safe and effective oral antibiotic for poultry, in addition to promoting the preservation of eggs and meat without causing adverse effects [22]. The first data on the beneficial application of *Syzygium aromaticum* essential oil (SAEO) for sanitizing hatching eggs have garnered attention from poultry researchers [18]. These benefits include a significant reduction in surface bacterial load, preservation of both the external and internal quality of eggs, and improvements in hatchability rates and post-hatch chick survival [18,23,24]. In addition, it did not have any adverse effects on the zootechnical performance of broiler chickens during the 70-day rearing period, including body weight, body weight gain, feed consumption, and feed conversion ratio [25]. Studies evaluating the safety of SAEO in the poultry field are still scarce. Moreover, to the best of our knowledge, no investigations have explored the impact of the topical application of SAEO-based sanitizers on hatching eggs and their association with bacterial counts in the yolk sac. Therefore, to prioritize the use of natural sanitizers in poultry farming and to further explore the potential of SAEO in managing hatching eggs, we evaluated the in vitro antibacterial activity of SAEO and investigated its effects on the bacterial load of eggshells and yolk sacs, as well as its toxicity profile.

## 2. Materials and Methods

The SAEO used for the in vitro antibacterial test [26] for spraying on eggshells and toxicity analysis [27] was supplied by Laszlo, Minas Gerais, Brazil. This oil, extracted through steam distillation from the leaves, was liquid, slightly yellow, and predominantly composed of eugenol (≈85.00%).

To identify an effective poultry sanitizing agent for hatching eggshells, we investigated the antibacterial properties of SAEO. The initial phase of our study focused on assessing its in vitro efficacy against bacteria commonly found on eggshells via the disk diffusion method. In this in vitro study, we evaluated the antibacterial efficacy of SAEO by applying 10 µL of the oil at serially diluted concentrations of 50 (500 mg/mL), 25, 12.50, 6.25, 3.13, 1.56, 0.78, 0.39, 0.20, and 0.10% (1.0 mg/mL) onto sterile disks. We prepared bacterial suspensions of *Pseudomonas aeruginosa* (*P. aeruginosa*) ATCC 27853 and *Staphylococcus aureus (S. aureus*) ATCC 25923 (American Type Culture Collection, Manassas, VA, USA), adjusted to the McFarland standard of 0.5. These suspensions were inoculated onto Mueller-Hinton agar plates, upon which the disks impregnated with the essential oil were placed. The plates were then incubated at 36 °C for 24 h. After the incubation period, we measured the diameters of the inhibition zones to assess the antibacterial efficacy of the disks. As controls, we used 30 µg of cefadroxil disks and 5% dimethyl sulfoxide solution. All concentrations of SAEO starting from 1.56% significantly inhibited *P. aeruginosa*, and all concentrations starting from 0.78% inhibited *S. aureus*. Therefore, the chosen concentration for the sanitizer formulation was 1.56%, which was capable of inhibiting both bacteria. The essential oil was stored under refrigeration (4 °C) until the preparation of the sanitizing solution and its application to the eggs.

To determine the mesophilic bacterial count on eggshells, hatching eggs from PSÇ (Pescoço Pelado Vermelho) chicken lineage breeders on a commercial farm were treated immediately after collection with sanitizing solution based on 1.56% SAEO (diluted in grain alcohol) or left untreated. For the treated eggs, approximately 2 to 3 mL of sanitizing solution was applied per egg using a manual sprayer. The ambient temperature at the time of application was approximately 22 °C. The eggs were dried at room temperature. One hour after the sanitization procedure, ten eggs per treatment were then placed in sterile bags (two per bag) (Labplas, Sainte-Julie, QC, Canada) containing 120 mL of 0.1% peptone saline solution (Laborclin, Pinhais, Paraná, Brazil) to wash the external surface. We then diluted the washing solutions to five times their initial volume using sterile saline solution. A 0.1 mL sample of each dilution was inoculated onto plate count agar (Ionlab, Araucária, Paraná, Brazil) and incubated for 48 h at 36 °C. After incubation, we counted the number of colonies formed and expressed the results as log_10_ values.

The remaining sanitized hatching eggs (*n* = 60) were stored for 24 h at approximately 20 °C and incubated to measure the bacterial load in the yolk sac, as previously described [28]. These hatching eggs were subjected to a 9-day incubation protocol in a single-stage setter (Premium Ecologica, Belo Horizonte, Minas Gerais, Brazil). During this period, the eggs were incubated at 37.7 °C with 60% relative humidity and turned automatically every hour. Sanitary conditions within the setter were ensured to minimize any significant external interference. Eggs without viable embryos were discarded, while those containing viable embryos (seven per treatment) were subjected to bacterial analysis.

We conducted the toxicity analysis using 60 non-sanitized hatching eggs from the same breeder lineage using the incubation procedure described above. Eggs without viable embryos were discarded, while those containing embryos underwent an incision in the air chamber region, followed by the removal of the shell and the underlying membrane. Each 200 µL solution (SAEO alone, SAEO combined with alcohol, or alcohol alone) was subsequently applied to the chorioallantoic membranes (CAMs) of 7 embryonated eggs. The presence or absence of lysis, hemorrhage, and coagulation on the CAM was observed over a period of 300 s, with measurements taken at 30, 120, and 300 s. The results were then classified on a scale ranging from non-toxic to severe toxicity [27].

After the results were tabulated in Excel (Microsoft 365, version 2404), the data were subjected to an analysis of variance (PROC GLM) and compared via the Tukey test, with a significance level of 5%. This analysis was conducted via SAS Studio University Edition software (SAS Institute Inc., Cary, NC, USA). *p* < 0.05 was considered significant.

## 3. Results and Discussion

**Figure 1:**
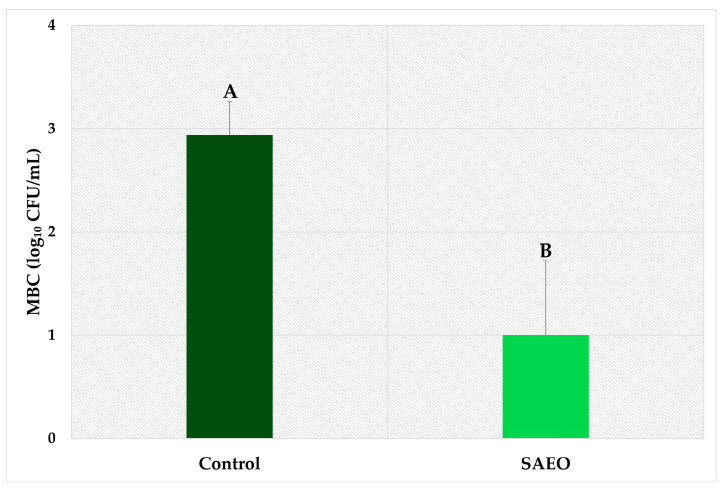
The mesophilic bacterial count (MBC) on treated and untreated eggshells. The treatment consisted of the application of *Syzygium aromaticum* essential oil (SAEO) at a concentration of 1.56%. The control group did not receive essential oil treatment. Bars represent the mean ± standard deviation of five repetitions. ^A,B^ Means with different letters are significantly different (*p* < 0.05).

To explore the bacterial susceptibility of eggshells to SAEO, we implemented a sanitization plan using this essential oil. Our bacterial count data revealed a notable reduction in bacterial load (Figure 1). Within just one hour after sanitization, we observed a reduction of almost 2.0 log_10_ in the mesophilic bacterial count on the eggshells (reduction from 2.94 ± 0.32 to 1.00 ± 0.72 log_10_ CFU/mL after sanitization with essential oil). Previous studies demonstrated reductions of 1.19 and 1.68 log_10_ in total aerobic mesophilic bacteria counts in eggshells after sanitization with SAEO at concentrations of 0.39% and 0.50%, respectively [18,23]. More recently, El-Soufi et al. [24] observed complete inhibition of bacterial growth on eggshells immediately after treatment with SAEO at a concentration of 0.39 mg/mL. The justification for this reduction is based on the proven antibacterial activity of SAEO [22], which manifests in a multisystemic way, mainly through the induction of bacterial apoptosis mediated by oxidative stress, followed by interference in DNA synthesis [22,29]. We did not evaluate the isolated effect of the alcohol used as a diluent for the essential oil, as its insignificant efficacy in bacterial reduction has been previously reported [18,28].

**Figure 2:**
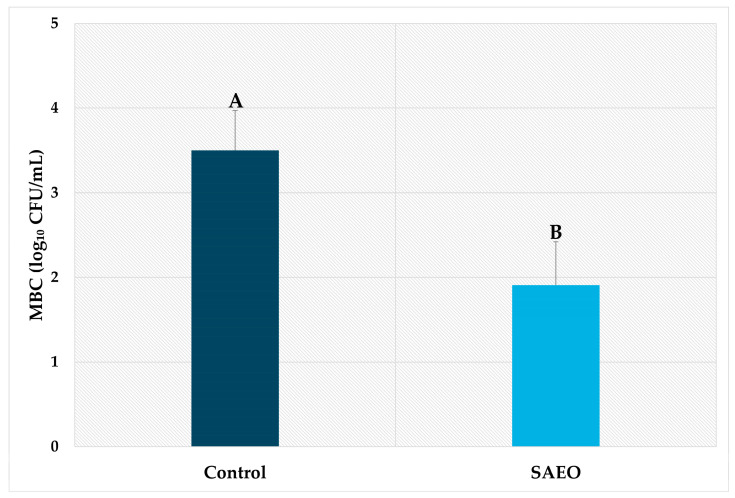
The mesophilic bacterial count (MBC) of yolk sacs from treated and untreated eggs. The treatment consisted of the application of *Syzygium aromaticum* essential oil (SAEO) at a concentration of 1.56%. The control group did not receive essential oil treatment. Bars represent the mean ± standard deviation of seven repetitions. ^A,B^ Means with different letters are significantly different (*p* < 0.05).

The yolk sac bacterial count data demonstrated a lower bacterial count in the yolk sac of eggs sanitized with SAEO (1.91 ± 0.51 log_10_ CFU/mL) compared to the control group (3.50 ± 0.47 log_10_ CFU/mL) (Figure 2). This difference corresponds to a reduction of more than 1.50 log_10_ in the bacterial load. Previously, we demonstrated that yolk sacs of eggs treated with essential oils of *Citrus aurantifolia*, *Ocimum basilicum*, and *Allium sativum* showed reductions of 52.65, 48.29, and 65.73%, respectively, in mesophilic bacterial populations compared to untreated eggs [28]. Furthermore, Franco et al. [30] reported that egg sanitization is associated with a lower bacterial load in the yolk sac. Our results can possibly be explained by the fact that when eggs do not undergo a sanitization process, the microbial load on the eggshell surface tends to be significantly high [31]. Although the shell has a physicochemical structure with antimicrobial properties, its porous nature may facilitate bacterial penetration, especially in cases of more intense contamination [32]. This risk is further heightened under favorable environmental conditions, such as during incubation, allowing bacteria to reach and colonize the embryos. Conversely, the use of effective sanitizers, such as essential oil, promotes a significant reduction in the bacterial load on the shell. This decrease may reduce the likelihood of microbial penetration and, consequently, the risk of embryonic contamination [28].

**Figure 3:**
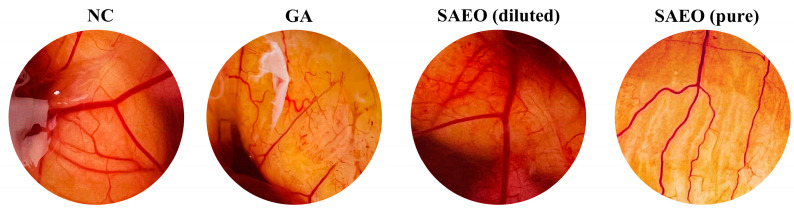
Photographic records of the results of the ‘hen’s egg test on the chorioallantoic membrane’ (HET-CAM) following the application of treatments. The photographs illustrate the effect of each treatment on blood vessel integrity. NC, negative control; GA, grain alcohol; SAEO, *Syzygium aromaticum* essential oil.

We conducted a toxicity experiment in which CAMs from developing embryos were exposed to aliquots of SAEO, both diluted and non-diluted, as well as to its carrier, grain alcohol. We observed that grain alcohol (GA) causes hemorrhage, coagulation, and lysis of the membrane immediately upon contact (Figure 3). On the other hand, SAEO showed moderate toxicity, as expected, as GA was used as the carrier for oil dilution. Recognizing the influence of the carrier, we decided to proceed with the experiment using pure SAEO. Notably, none of the CAMs exposed to pure SAEO exhibited any abnormalities during the five minutes of contact. Therefore, GA, which is used as an essential oil diluent for sanitizing hatching eggs, must be applied exclusively topically to the eggshell. Using the same toxicity assessment model and corroborating our findings, Racea et al. [33] reported that 1 mM eugenol, the main compound in SAEO, had low irritation potential on the CAM. Additionally, Silva et al. [34] reported that a 1% aqueous extract of *Syzygium aromaticum* did not cause irritation to the CAM.

## 4. Conclusions

This study highlights the challenging relationship between bacterial contamination and the management of sanitizing hatching eggs. Although we did not focus primarily on production rates, our research provides significant insights into the role of SAEO as an effective sanitizer. Our results indicate that SAEO has a favorable safety profile for topical use on eggshells, demonstrating its beneficial capacity in reducing bacteria present on eggshells and yolk sacs. It is crucial to conduct a cost assessment and economic feasibility study of the proposed sanitization method.

## Data Availability

The data are contained within the article.
